# The dual role of VEGF-A in a complex *in vitro* model of oxaliplatin-induced neurotoxicity: Pain-related and neuroprotective effects

**DOI:** 10.1016/j.neurot.2025.e00532

**Published:** 2025-02-12

**Authors:** A. Toti, E. Lucarini, V. Ferrara, C. Parisio, C. Ciampi, E. Gerace, L. Micheli, F. Margiotta, D. Venturi, T. Mello, P.M. Lacal, G. Graziani, G. Mannaioni, C. Ghelardini, L. Di Cesare Mannelli

**Affiliations:** a Department of Neuroscience, Psychology, Drug Research and Child Health - NEUROFARBA - Pharmacology and Toxicology Section, University of Florence, Florence, Italy; b Department of Health Sciences, University of Florence, Florence, Italy; c Department of Experimental and Clinical Biomedical Sciences “Mario Serio”, University of Florence, Florence, Italy; d Laboratory of Molecular Oncology, IDI-IRCCS, Rome, Italy; e Department of Systems Medicine, Pharmacology Section, University of Rome Tor Vergata, Rome, Italy

**Keywords:** Chemotherapy-induced neurotoxicity, Spinal cord organotypic slices, Glial cells, VEGF-A, Neuroprotection, VEGFRs

## Abstract

Vascular endothelial growth factor (VEGF)-A is a main player in the development of neuropathic pain induced by chemotherapy and the pharmacological blockade of VEGF receptor (VEGFR) subtype 1 is a pain killer strategy. Interestingly, VEGF-A has been demonstrated to have also neuroprotective properties. The aim of the study was to investigate the neuroprotective role of VEGF-A against oxaliplatin neurotoxicity, attempting to discriminate pain-related and restorative signaling pathways. We used rat organotypic spinal cord slices treated with oxaliplatin, as an *in vitro* model to study chemotherapy-induced toxicity*.* In this model, 10 ​μM oxaliplatin caused a time-dependent release of VEGF-A, which was reduced by the astrocyte inhibitor fluorocitrate. Moreover, glia inhibition exacerbated oxaliplatin-induced cytotoxicity in a VEGF-A sensitive manner. Treatment with VEGF_165_b, the main isoform of VEGF-A, prevented the oxaliplatin-induced neuronal damage (indicated by NeuN staining) and astrocyte activation (indicated by GFAP staining). In addition, the blockade of VEGFR-2 by the selective antibody DC101 blunted the protective action of VEGF_165_b. In the same model, VEGF_165_b increased the release of molecules relevant in pain signaling, like substance P and CGRP, as well as the mRNA expression of glutamate transporters (EAAT1 and EAAT2), similarly to oxaliplatin and these effects were prevented by the selective VEGFR-1 blocker antibody D16F7. In conclusion, VEGF-A plays a dichotomic role in an *in vitro* model of chemotherapy-induced toxicity, either promoting neuroprotection or triggering pain mediators release, depending on which of its two receptors is activated. The selective management of VEGF-A signaling is suggested as a therapeutic approach.

## Graphical abstract

**Figure:**
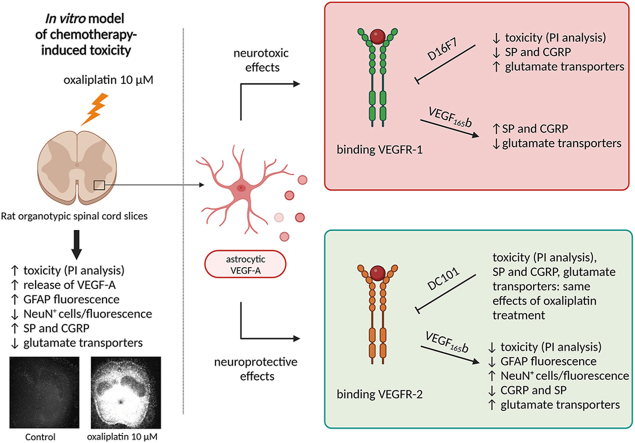

## Background

Neuropathic pain can arise as a consequence of various pathologies such as diabetes, infection, nerve injury or cancer [1]. In particular, 68 ​% of cancer patients affected treated with taxanes, platinum compounds and vinca alkaloids develop chemotherapy-induced neuropathy (CIN) within the first month, 60 ​% within three months, and 30 ​% within six months of treatment start [2]. This important adverse effect may require dose reduction or interruption of drug administration, leading to suboptimal cancer treatment. The mechanisms underlying the development of neuropathic pain are not yet fully understood, highlighting the need for further research. Alongside neurons, glial cells such as astrocytes, have recently emerged as key players in pain mediation. Astrocytes are the most abundant cells in central nervous system (CNS) [3] and they cover various roles such as glutamate detoxification, water and ions homeostasis maintenance, control of blood-brain barrier permeability, trophic and metabolic support to neurons [[4], [5], [6]].

During a pathological condition, astrocytes can became activated and induce astrogliosis by releasing mediators such as cytokines [e.g., tumor necrosis factor-α (TNF-α), interleukin 1 β (IL-1β)], chemokines [e.g., monocyte chemoattractant protein-1 (MCP-1/CCL2) and the chemokine (C-X-C motif) ligand 1 (CXCL1)], growth factors [e.g., brain-derived neurotrophic factor (BDNF), basic fibroblast growth factor (bFGF) and vascular endothelial growth factor-A (VEGF-A)] and proteases [e.g., matrix metalloproteinase-2 (MMP-2) and tissue-type plasminogen activator (tPA)], together with gliotransmitters such as glutamic acid and adenosine triphosphate (ATP), which are linked to the onset and maintenance of pain [7].

Among the plethora of cytokines released, VEGF-A is emerging as an important player in various processes involving the CNS, in addition to the well-known vascular properties [8,9].

Recent studies have demonstrated an increased plasma level of VEGF-A in oxaliplatin-induced neuropathic mice, highlighting an involvement of this factor in pain mediation [10].

VEGF-A exerts its function by binding two related tyrosine kinase receptors, VEGFR-1 and VEGFR-2. Both receptors carry seven immunoglobulin-like domains in the extracellular region, a single transmembrane region and a consensus tyrosine kinase sequence that is interrupted by a kinase-insert domain [11,12]. VEGFR-1 is mainly expressed by activated astrocytes and microglia after acute injury, whereas VEGFR-2 is expressed in several neuron populations and in some glial cells [[13], [14], [15], [16]]. VEGFR-2 is known for its contribution in physiological angiogenesis [17] and in neuroprotective pathways [[18], [19], [20], [21]] while VEGFR-1 has been associated with pathological processes such as inflammation, tumor-associated angiogenesis [22] and tumor progression, being up-regulated in a variety of cancers [23].

We have previously shown that the monoclonal antibody (mAb) D16F7, a specific blocker of VEGFR-1, was able to attenuate the neuropathic pain induced by neurotoxic anticancer drugs [24]. Moreover, silencing of VEGFR-1 in astrocytes of the spinal cord dorsal horn, strongly reduced the onset of pain after oxaliplatin administration [24].

Here, we examined the bifunctional role of VEGF-A as both pain-related or protective player in an *in vitro* model of oxaliplatin-induced neurotoxicity, using rat organotypic spinal cord culture, a model that well captures the complexity of CNS.

## Material and Methods

### Study approval

All animal manipulations were carried out at CeSAL (Centro Stabulazione Animali da Laboratorio, University of Florence) according to the Directive 2010/63/EU of the European parliament and of the European Union council (September 22, 2010) on the protection of animals used for scientific purposes and with IASP. The ethical policy of the University of Florence complies with the Guide for the Care and Use of Laboratory Animals of the US National Institutes of Health (NIH Publication No. 85–23, revised 1996; University of Florence assurance number: A5278- 01). Formal approval to conduct the experiments described was obtained from the Italian Ministry of Health (No. 17.E9C.N.NOV), from the Animal Subjects Review Board of the University of Florence and from the Animal Ethics Committee of University of Campania of Naples. Experiments involving animals have been reported according to ARRIVE guidelines [25]. All efforts were made to minimize animal suffering and to reduce the number of animals used.

### Preparation of rat organotypic spinal cord slice cultures

Organotypic spinal cord slice cultures were setup according to the previously reported method, with minor modifications [26,27]. Briefly, 4- to 6-day old male and female pups from Sprague-Dawley pregnant rats (Envigo, Varese, Italy) were used for the experiments. Spinal cord was isolated from rat pups and transverse slices (420 ​μm) were prepared using a McIlwain tissue chopper from all the section of spinal cord (sacral, lumbar, thoracic, and cervical). The slices were then transferred onto 30 ​mm diameter semiporous membranes inserts (Millicell-CM PICM03050; Millipore, Italy) (five slices for each insert), which were placed in six well tissue culture plates containing 1.2 ​mL medium per well (Suppl. File 1a). The culture medium consisted of 50 ​% Eagle's minimal essential medium, 25 ​% heat-inactivated horse serum, 25 ​% Hanks' balanced salt solution, 5 ​mg/mL glucose, 2 ​mM l-glutamine, 3.75 ​mg/mL amphotericin B, 1 ​% of penicillin (100 U/mL) and streptomycin (100 ​μg/mL). The culture medium was changed three times a week. Slices were maintained at 37 ​°C in an incubator in atmosphere of humidified air and 5 ​% CO_2_ for 14 days. During their maturation *in vitro*, the slices reduce their initial thickness but maintain their original structural and integrity, as shown in the Suppl. File 1b. Before experiments, all the slices were screened for viability by phase-contrast microscopy analysis; slices displaying signs of neurodegeneration were discarded from the study (exclusion criteria).

### Oxaliplatin-induced neurotoxicity protocol in rat organotypic spinal cord slices

To simulate chemotherapy-induced neurotoxicity *in vitro,* cultured slices were exposed to increasing concentrations of oxaliplatin (1, 10 and 100 ​μM) at different times (6 and 24h). Cell injury was evaluated by analyzing the fluorescence of propidium iodide (PI, 5 ​μg/mL; Merck, Milan, Italy. Cod. 81845), as reported in the paragraph below. To investigate the neuroprotective role of VEGF-A, all tested agents were added to the culture medium alone or in combination with oxaliplatin and were kept in culture until neuronal injury was evaluated 24 ​h later.

### Assessment of spinal cord cell injury assay

For toxicity analysis, 5 ​μg/mL PI was added to the medium and 30 ​min later, fluorescence was evaluated using an inverted fluorescence microscope (Olympus IX-50; Solent Scientific, Segensworth, United Kingdom) equipped with a xenon-arc lamp, a low-power objective (4×) and a rhodamine filter [26]. Images were digitalized by using a video image obtained by a CCD camera (Diagnostic Instruments Inc., Sterling Heights, MI, USA), controlled by InCyt Im1TM software (Intracellular Imaging Inc., Cincinnati, OH, USA) and subsequently analyzed using the Image-Pro Plus morphometric analysis software (Media Cybernetics, Silver Spring, MD, USA). To quantify cell death, the slice was identified and encompassed in a frame using the drawing function in the image software (ImageJ; NIH, Bethesda, MD, USA) and the optical density of PI fluorescence was recorded.

### Immunofluorescence staining in free-floating

Organotypic spinal cord slices were fixed with cold 4 ​% paraformaldehyde solution at 4 ​°C overnight and stained according to Croft et al. [28,29]. Briefly, formalin-fixed slices were washed with cold Phosphate Buffered Saline (PBS) 1X and then removed and transferred into 24-multiwell plate with 1 ​mL of PBS 1X. The slices were incubated with blocking solution (PBS, 0.3 ​% Triton X-100, 5 ​% albumin bovine serum; PBST), at room temperature for 1 ​h and were subsequently probed with the primary antibodies anti-glial fibrillary acidic protein (GFAP) (GeneTex, Irvine, CA, USA; GTX108711, Lot:44258 diluition 1:500), and anti-RECA-1 (Bio-Rad, Hercules, CA, USA MCA970GA, Lot:149443, diluition 1:200) in PBST overnight at 4 ​°C. The day after, slices were washed three times with PBS for 10 ​min and immunodetection was performed with secondary antibodies (goat anti-rabbit or anti-mouse IgG) labeled with Alexa Fluor 568 (Life Technologies, Rockford, IL, USA, diluition 1:500) and Alexa Fluor 488- conjugated antibodies (for NeuN staining, Millipore, Milan, Italy; MAB377X, Lot:3117794, diluition 1:100), in incubation buffer at room temperature. The slices were finally incubated with DAPI (500 ​ng/mL), a nuclear-marker, and were mounted on slides using Fluoromount™ (Life Technologies, Rockford, IL, USA). Images were acquired using a motorized Leica DM6000 B microscope equipped with a DFC350FX camera (Leica, Mannheim, Germany). For the fluorescence intensity analysis of GFAP and NeuN, two different images for each slice were acquired, for a total of 12 slices, collected through a 20x objective. For NeuN^+^ cell count, four different images for each slice were acquired, for a total of 12 slices, collected through a 40x objective. For RECA-1 staining, the same region of 10 slices per condition were analyzed using the Simple Neurite Tracer plugin in ImageJ to measure the total length of the vasculature.

### Evaluation of VEGF-A and neuropeptides in culture medium

At the end of the experiments, culture medium was collected and used to measure the concentrations of VEGF-A, substance P (SP) and calcitonin gene-related peptide (CGRP), assessed by enzyme-linked immunosorbent assay (ELISA) kit (according to manufacturer's instructions: ThermoFisher, Waltham, MA, USA, ERVEGFA for VEGF-A; MyBioSource, San Diego, CA, USA, MBS703659 for SP; BIOMATIK, Kitchener, Canada, EKF58049 for CGRP). The results were normalized to cell protein concentration and expressed as percentage of the values obtained from control samples.

### RNA isolation, reverse transcription, and real time polymerase chain reaction (RT-PCR)

Total RNA was isolated from rat organotypic spinal cord slices using TRI Reagent (Merck, Milan, Italy). One microgram of RNA was retrotranscribed using PrimeScript™ RT reagent Kit with gDNA eraser (Takara Bio, Kusatsu, Shiga, Japan, cat#RR047A). Real Time-PCR (RT-PCR) was performed using SsoAdvanced Universal SYBR® Green Supermix (Bio-Rad, Milan, Italy). The following primers were used: EAAT1: forward 5′- CAGTCATCGTCGGCCTCCTCATTC -3′ and reverse 5′- CTGGTGATGCGTTTGTCCACACCATTG -3′ (Invitrogen, Milan, Italy); validated primers for rEAAT2 and rGAPDH were purchased from Bio-Rad (qRnoCED0005967 and qRnoCID0057018). The calculation of differential expression of the transcripts was performed by the 2^−ΔΔCt^ formula and normalized using the GAPDH reference gene.

### Statistics

Results were expressed as mean ​± ​S.E.M of three experiments from independent cell preparations. The analysis of variance was performed by ONE-way ANOVA followed by Bonferroni post-hoc comparisons test. P values less than 0.05 were considered significant. Data were analyzed by using the “Origin® 10” software.

## Results

### Structural organization and vascular network in mature organotypic spinal cord slices

**Fig. 1:**
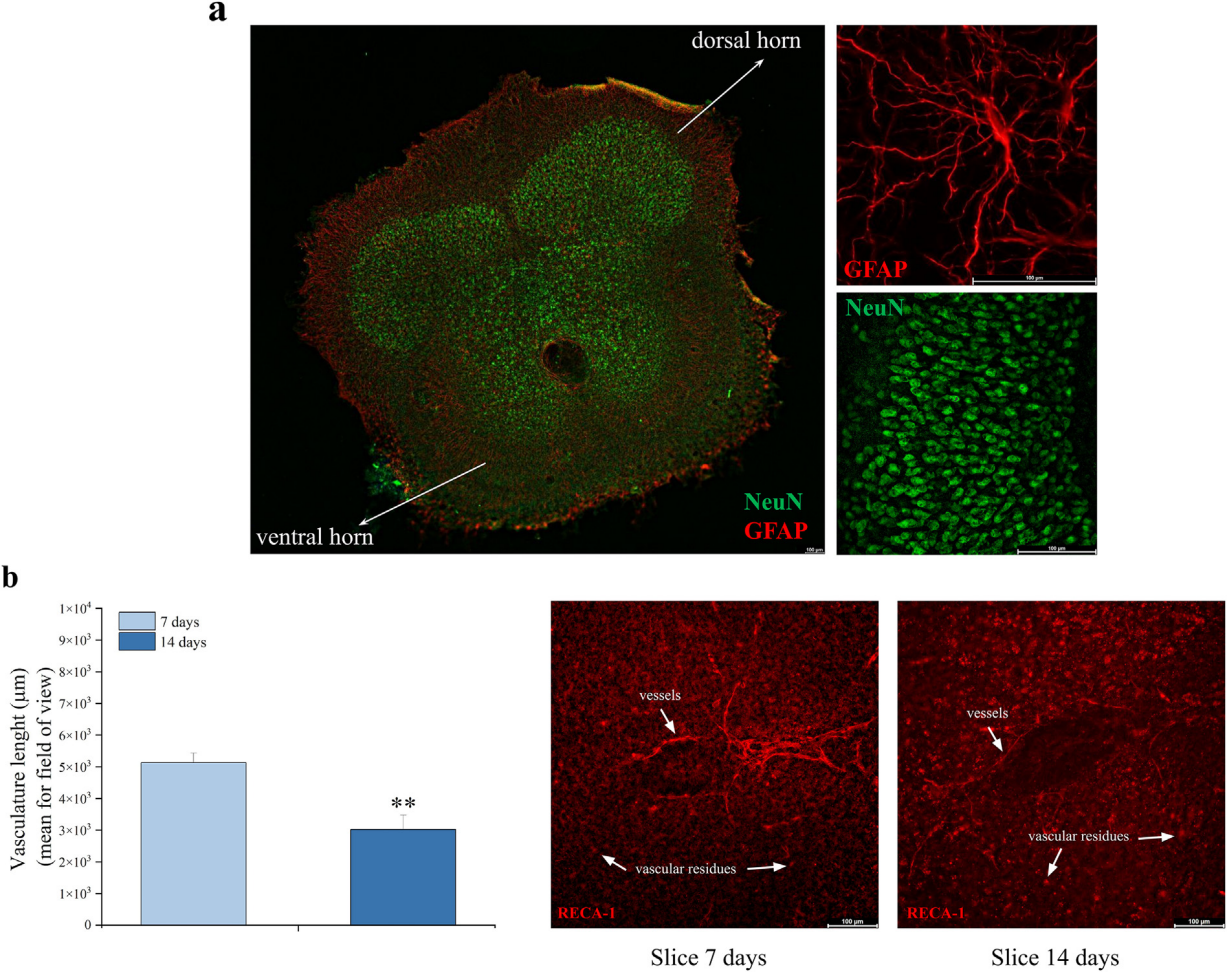
**Structural organization and vascular network in organotypic spinal cord slices.** (a) Immunohistochemical analysis of GFAP (astrocyte marker, red) and NeuN (neuron marker, green) performed after 14 days of slice maturation (image obtained by scanning with 20X magnification of each field of view; 63× magnification for GFAP insert and 40× magnification for NeuN inserts). Scale bar ​= ​100 ​μM. (b) Immunohistochemical analysis and representative images of RECA-1 (blood vessels marker, red), performed after 7 and 14 days of slices maturation (40X magnification). Scale bar ​= ​100 ​μM ∗∗P ​< ​0.01 vs slice 7 days.

Organotypic spinal cord slices represent an important crossroad between *in vitro* and *in vivo* studies. In this work, we used spinal cord cultures to investigate the non-vascular role of VEGF-A in nervous tissue and its functions in CIN. To characterize spinal cord tissue organization after 14 days, we analyzed the slices by immunofluorescence, using GFAP (for astrocyte cells) and NeuN (for neuronal cells) markers. As shown in Fig. 1a, cell morphology and structural organization of the spinal cord were preserved in the slices after 14 days in culture. Furthermore, by using the endothelial marker RECA-1, we quantify the length of the vasculature, highlighting a significant reduction of the normal vascular network during maturation (day 14 *vs* day 7, Fig. 1b), a fundamental aspect that allowed us to study the neural VEGF-A.

### Oxaliplatin induces time- and concentration-dependent toxicity in organotypic spinal cord slices

**Fig. 2:**
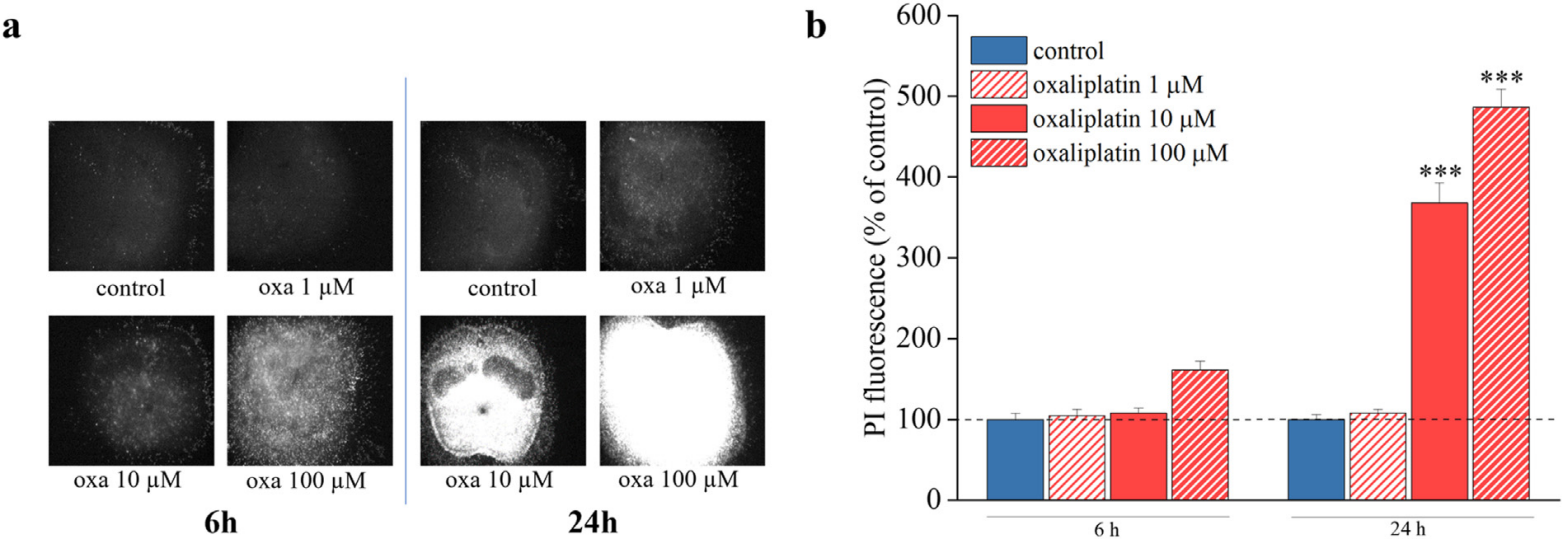
**Oxaliplatin induces cytotoxicity in spinal cord slices.** Qualitative (a) and quantitative (b) analyses of PI (5 ​μg/mL) fluorescence intensity were performed to investigate the toxicity of oxaliplatin at different concentrations (1, 10 and 100 ​μM) for 6 and 24 ​h. Each value represents the mean ​± ​SEM of three independent experiments, with a total of 15 slices for condition. ∗∗∗P ​< ​0.001 vs control.

After 14 days in culture, organotypic spinal cord slices were treated with increasing concentrations of oxaliplatin (1, 10 and 100 ​μM) for 6 and 24 ​h (Fig. 2), and toxicity was evaluated by PI staining (PI, 5 ​μg/mL). Quantitative analysis of PI fluorescence showed that treatment with 10 ​μM oxaliplatin for 24 ​h induced a statistically significant increase of neurotoxicity compared to control slices (Fig. 2a and b). Moreover, 10 ​μM oxaliplatin did not result in a complete damage of the tissue, as observed with 100 ​μM (as shown in Fig. 2a and b). Therefore, 10 ​μM oxaliplatin was chosen for all the subsequent experiments.

**Fig. 3:**
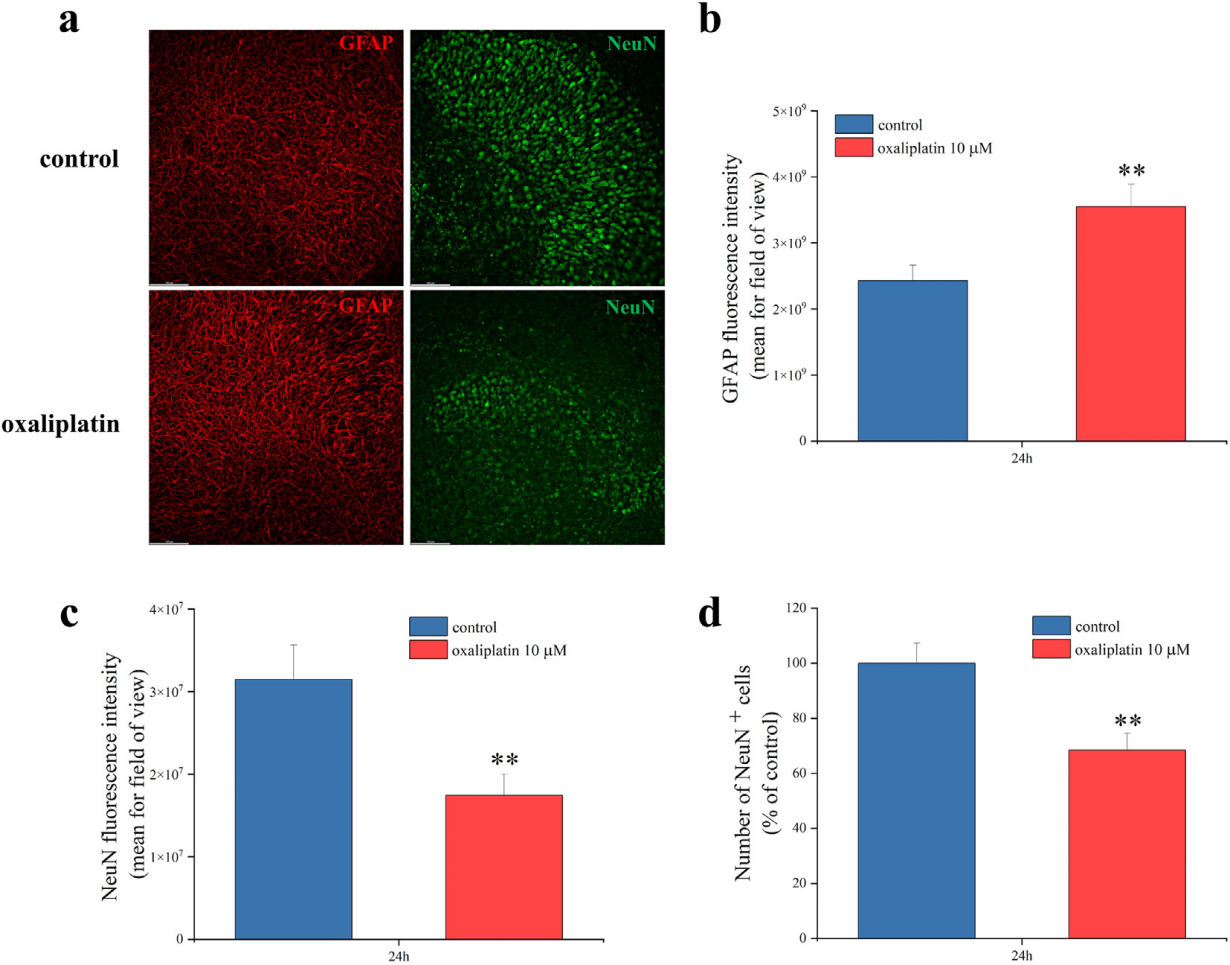
**Alterations of astrocytes and neurons induced by oxaliplatin.** (a) Representative images of GFAP (red) and NeuN (green) staining in organotypic spinal cord slices, treated with 10 ​μM oxaliplatin for 24 ​h in comparison to untreated slices. Quantitative analyses were expressed as mean of GFAP fluorescence intensity (b), NeuN fluorescence intensity (c) and NeuN positive cells per optic field (d) (20X magnification). Scale bar = 100 μM. Each value represents the mean ​± ​SEM of three independent experiments, with a total of 12 slices for condition. ∗∗P ​< ​0.01 vs control.

To characterize the alterations induced by oxaliplatin in spinal cord slices, we performed immunofluorescence experiments evaluating astrocytes (GFAP staining, red) and neurons (NeuN staining, green). As shown in Fig. 3, Fig. 10, 10 μM oxaliplatin for 24 ​h induced a statistically significant increase of the GFAP fluorescence intensity compared to the untreated control slices (Fig. 3a left panels and 3b). In addition, oxaliplatin caused alterations of neurons (Fig. 3a right panels), with a reduction in the NeuN fluorescence intensity (Fig. 3c) as well as in the number of NeuN^+^ cells (Fig. 3d).

### Inhibition of glial metabolism blocks oxaliplatin-induced release of VEGF-A, causing cytotoxicity

**Fig. 4:**
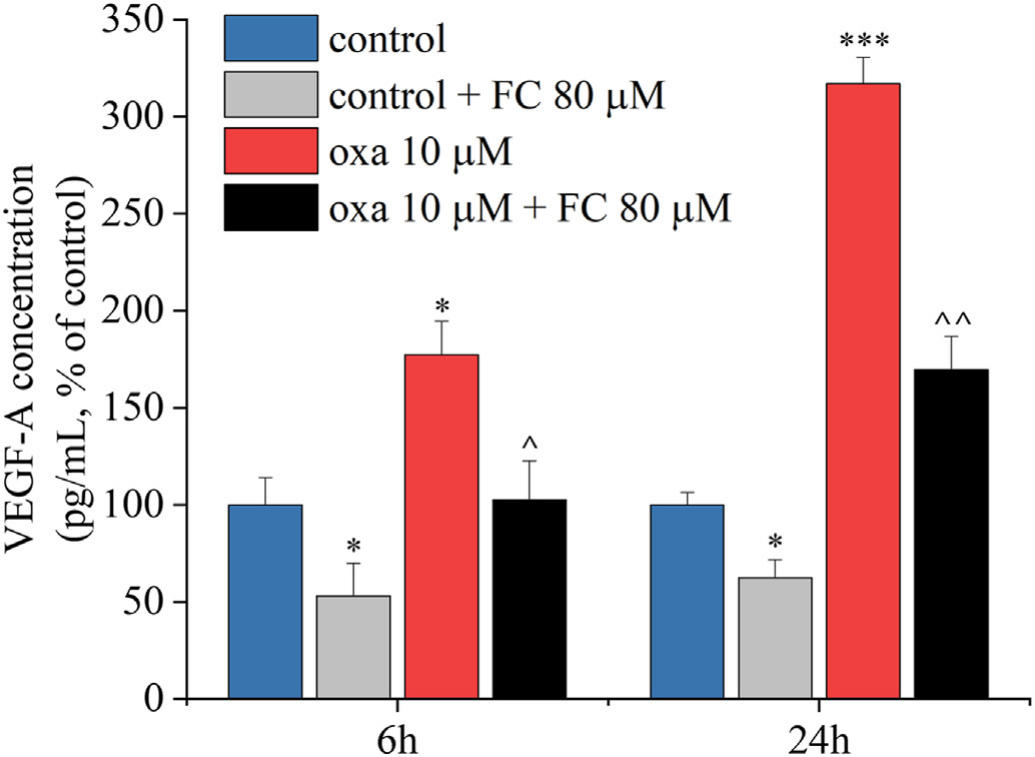
**Fluorocitrate inhibits oxaliplatin-induced release of VEGF-A.** Amount of VEGF-A measured by ELISA into the culture medium of the organotypic slices, after treatment with 80 ​μM fluorocitrate (FC) alone or in combination with 10 ​μM oxaliplatin (oxa) for 6 and 24 ​h. VEGF-A levels were normalized to cell protein concentrations. Each value represents the mean ​± ​SEM of three independent experiments, with two wells per condition. ∗P < 0.05 and ∗∗∗P < 0.001 vs control; ˆ P < 0.05 and ˆ ˆ P < 0.01 vs oxaliplatin treatment.

We assessed the release of VEGF-A in the culture medium of spinal cord slices treated with 10 ​μM oxaliplatin after 6 and 24 ​h by ELISA. Our results show that oxaliplatin exposure induced a statistically significant and time-dependent increase of VEGF-A that was blunted by the inhibitor of glial cell metabolism fluorocitrate (FC, 80 ​μM), both in control and oxaliplatin treated slices (Fig. 4), suggesting that VEGF-A is mainly produced by glial cells.

**Fig. 5:**
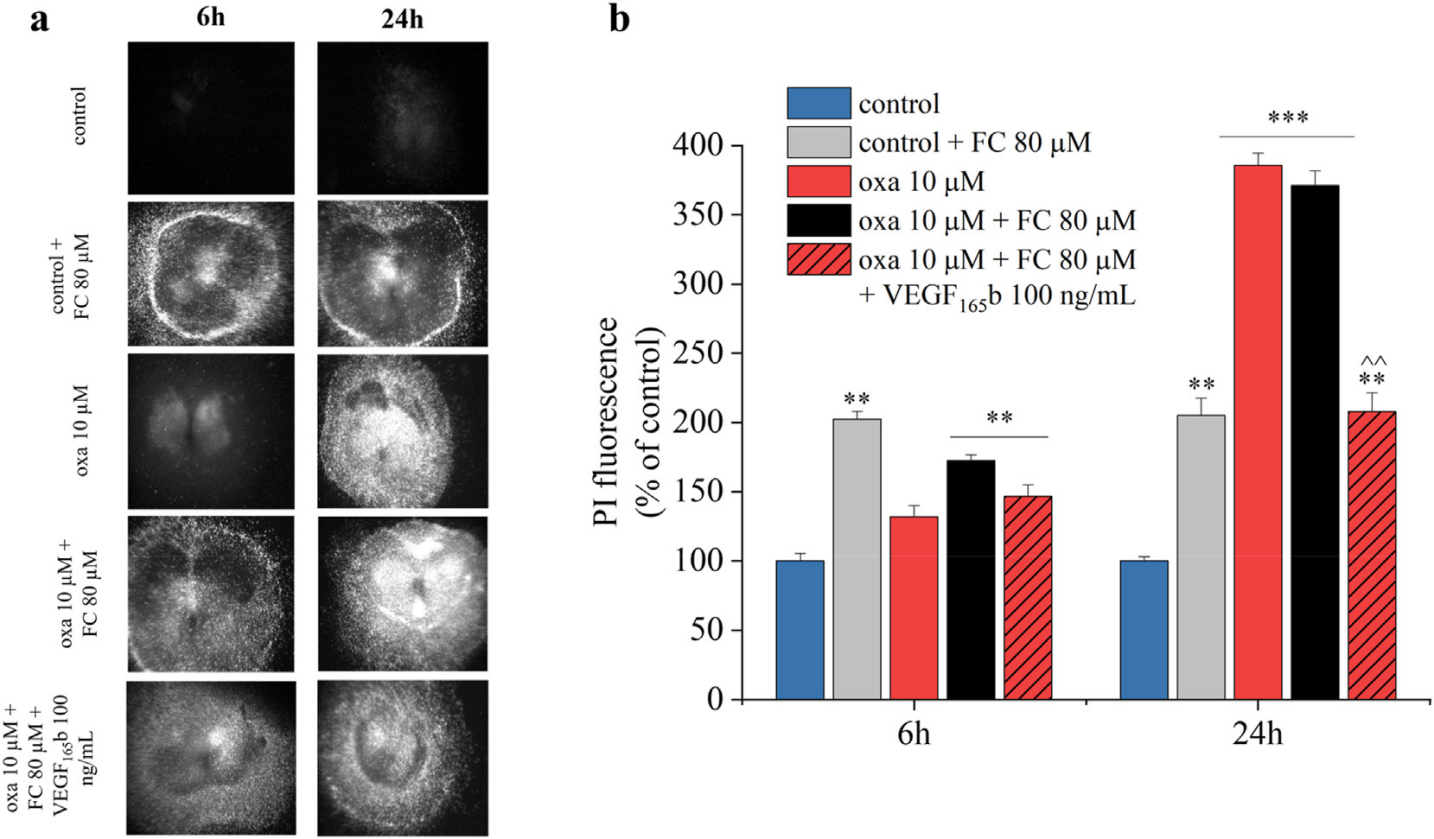
**Inhibition of astrocytic VEGF-A by fluorocitrate enhances oxaliplatin cytotoxicity.** Qualitative (a) and quantitative (b) analysis of PI (5 ​μg/mL) fluorescence intensity in spinal cord slices to evaluate the effect of 80 ​μM fluorocitrate (FC) alone or in combination with 100 ​ng/mL VEGF_165_b on 10 ​μM oxaliplatin-induced toxicity applied for 6 and 24 ​h. Each value represents the mean ​± ​SEM of three independent experiments, with a total of 15 slices for condition. ∗∗P ​< ​0.01 and ∗∗∗P ​< ​0.001 vs control; ˆ ˆ P ​< ​0.01 vs oxaliplatin treatment.

The addition of FC to the culture medium caused toxicity in control slices at any time considered (6 and 24 ​h) (Fig. 5a–b, grey column). The concomitant application of fluorocitrate (80 ​μM) together with oxaliplatin (10 ​μM) induced a toxicity that is prevented by the addition of exogenous VEGF_165_b (100 ​ng/mL, 24h), the main isoform of VEGF-A, suggesting the neuroprotective action of VEGF-A (Fig. 5a–b).

### VEGF_165_b exerts neuroprotective effects on oxaliplatin-induced cytotoxicity

**Fig. 6:**
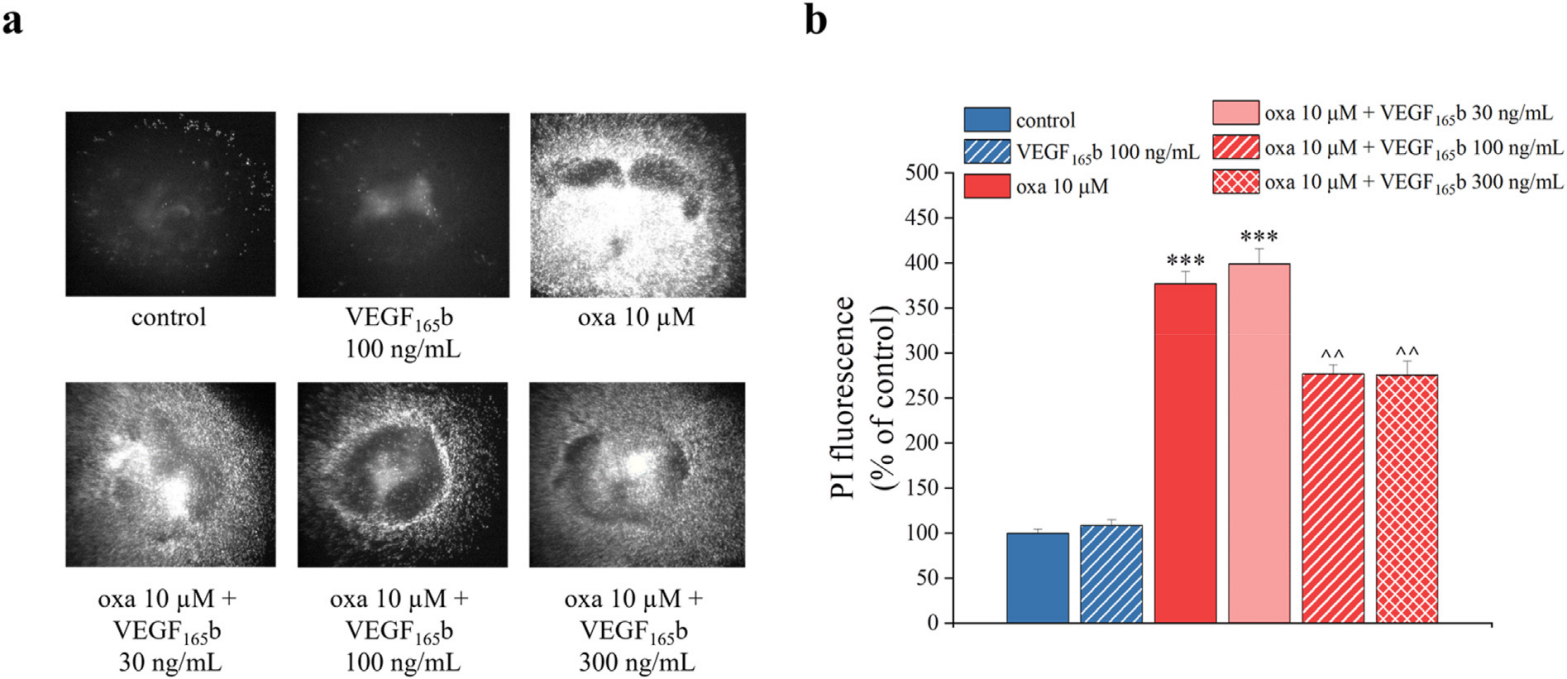
**VEGF**_**165**_**b prevents the cytotoxicity induced by oxaliplatin.** Qualitative (a) and quantitative (b) analyses of PI (5 ​μg/mL) fluorescence intensity were performed to study the effect of VEGF_165_b treatment (30, 100 and 300 ​ng/mL) on 10 μM oxaliplatin-induced toxicity at 24 ​h. Each value represents the mean ​± ​SEM of three independent experiments, with a total of 15 slices for condition. ∗∗∗P ​< ​0.001 vs control; ˆ ˆ P ​< ​0.01 vs oxaliplatin treatment.

To address the neuroprotective role of VEGF_165_b observed above, spinal cord slices were co-treated with 10 ​μM oxaliplatin and VEGF_165_b (30, 100 and 300 ​ng/mL) for 24 ​h. VEGF_165_b alone did not produce a significant effect in the control conditions, however it clearly reduced the oxaliplatin-induced toxicity in a dose-dependent manner, as shown in Fig. 6a and b.

**Fig. 7:**
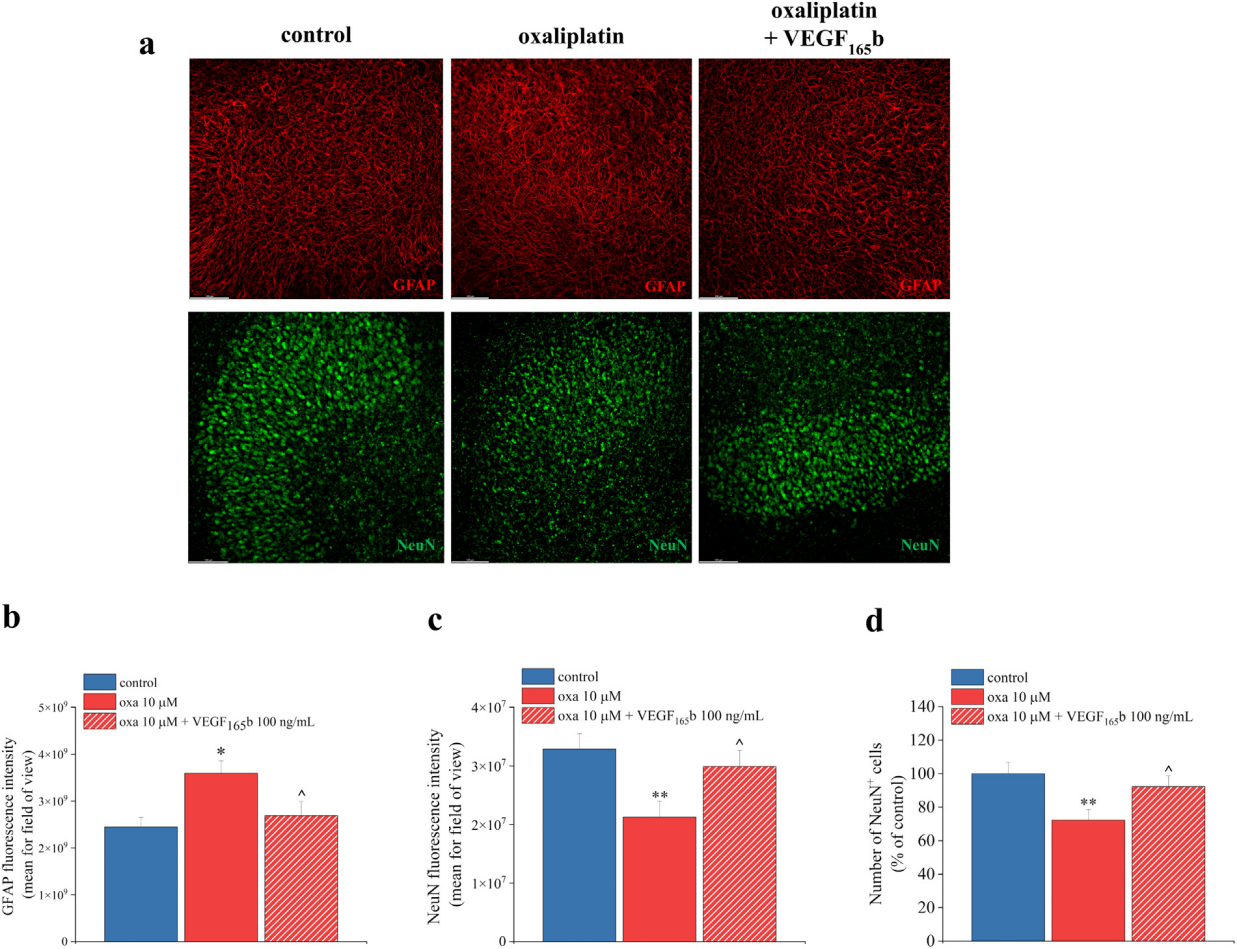
**VEGF**_**165**_**b reduces the alterations in astrocytes and neurons caused by oxaliplatin.** Representative images of GFAP (red) and NeuN (green) staining on organotypic spinal cord slices, treated with oxaliplatin (10 ​μM) alone or in combination with VEGF_165_b (100 ​ng/mL) for 24 ​h in comparison to untreated slices (a). Quantitative analyses were expressed as mean of GFAP fluorescence intensity (b), NeuN fluorescence intensity (c) and NeuN positive cells per optic fields (d). (20X magnification). Scale bar = 100 μM. Each value represents the mean ​± ​SEM of three independent experiments, with a total of 12 slices for condition. ∗P ​< ​0.05 and ∗∗P ​< ​0.01; ˆ P ​< ​0.05 vs oxaliplatin treatment.

Moreover, to observe the effect of VEGF_165_b on oxaliplatin-induced alterations, we evaluated the variations in astrocytes and neurons after co-treatment with 100 ​ng/mL VEGF_165_b and 10 μM oxaliplatin for 24 h. We observed a statistically significant reduction in the GFAP fluorescence intensity in the slices co-treated with oxaliplatin and VEGF_165_b 100 ​ng/mL, compared to those treated with oxaliplatin alone (Fig. 7a upper panels and 7b); VEGF_165_b 100 ​ng/mL was also able to reduce the neuronal loss caused by oxaliplatin, as indicated by the NeuN fluorescence (Fig. 7a lower panels and 7c) and the number of NeuN^+^ cells (Fig. 7d).

### Molecular mechanism underlying VEGF-A-mediated neuroprotection

**Fig. 8:**
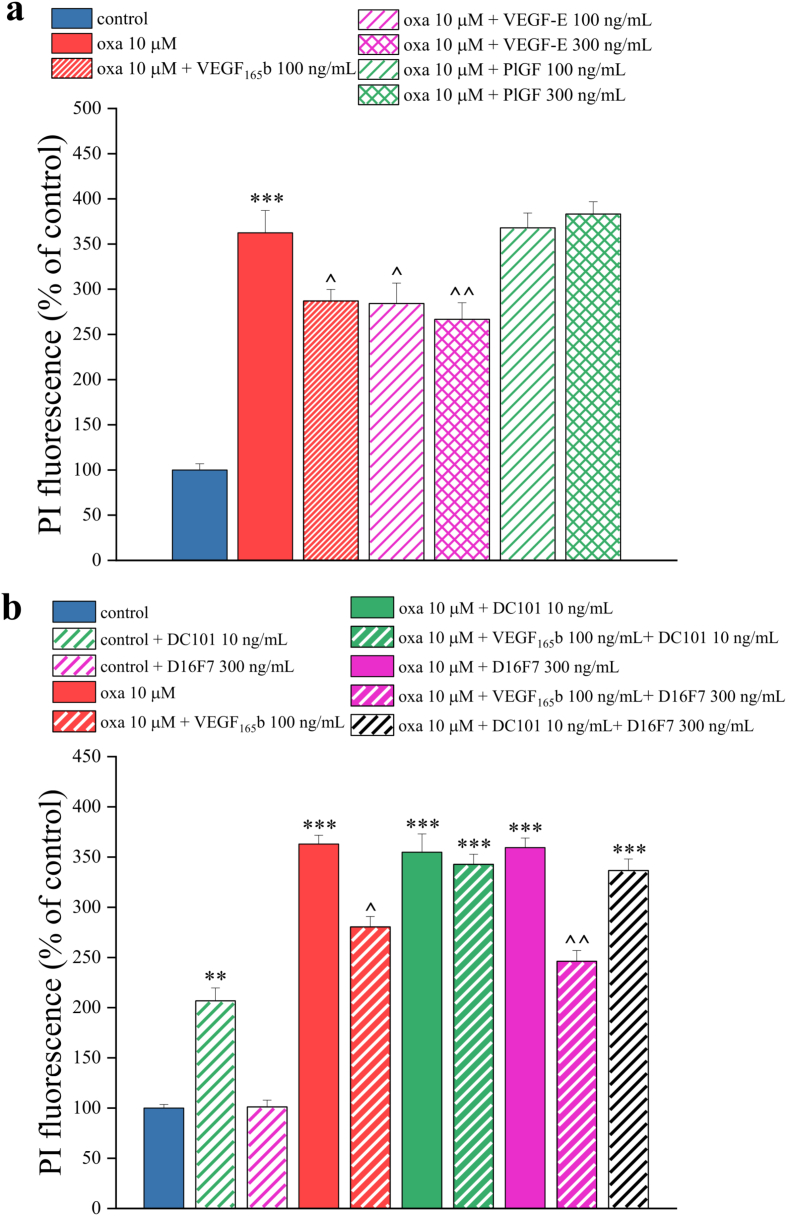
**VEGFR-2 mediates the VEGF-A neuroprotective effects.** Quantitative analyses of PI (5 ​μg/mL) fluorescence intensity performed to evaluate the effect of 24 ​h treatment with the VEGFR-2 ligand, VEGF-E (100 and 300 ​ng/mL), and the VEGFR-1 ligand, PlGF (100 and 300 ​ng/mL), (a) and with the selective VEGFR-1 antagonist, D16F7 (300 ​ng/mL) or the selective VEGFR-2 antagonist, DC101 (10 ​ng/mL) mAbs (b) on oxaliplatin-induced toxicity (10 ​μM, 24 ​h), in combination with VEGF_165_b (100 ​ng/mL). Each value represents the mean ​± ​SEM of three independent experiments. ∗∗P ​< ​0.01; ∗∗∗P ​< ​0.001 vs control; ˆ P ​< ​0.05; ˆ ˆ P ​< ​0.01 vs oxaliplatin treatment.

To investigate the molecular mechanisms underlying VEGF-A-mediated neuroprotection, the spinal cord slices were treated for 24 ​h with oxaliplatin (10 ​μM) in combination with placental growth factor (PlGF, 100 ​ng/mL and 300 ​ng/mL) or VEGF-E (100 ​ng/mL and 300 ​ng/mL), selective ligands for VEGFR-1 and VEGFR-2, respectively. As show in Fig. 8a (and Suppl. File 2), we found that the selective activation of VEGFR-2 by VEGF-E reduced the oxaliplatin-induced toxicity in a concentration-dependent manner, an effect that was not observed for the activation of VEGFR-1 by P1GF. Collectively, these results strongly suggest that VEGF-A-induced neuroprotection might be mediated by its binding to VEGFR-2.

To confirm this hypothesis, we tested two specific receptor blockers, D16F7 (300 ​ng/mL, an anti-VEGFR-1 mAb) and DC101 (10 ​ng/mL, an anti-VEGFR-2 mAb) alone or in combination with oxaliplatin (10 ​μM) for 24 ​h. We observed that DC101, but not D16F7, caused toxicity in the control slices, confirming that VEGFR-2 plays a crucial role in maintaining the physiological state of the slices. Conversely, treatment with DC101 and D16F7, alone or in combination, did not worsen the toxic effects of 10 ​μM oxaliplatin (Fig. 8b and Suppl. File 3). Interestingly, DC101 reverted the neuroprotective effect mediated by VEGF_165_b on oxaliplatin-treated slices (Fig. 8b and Suppl. File 3). Taken together, these results confirmed that VEGF-A also plays a neuroprotective role, mediated by the VEGFR-2 receptor.

### The anti-VEGFR-1 mAb D16F7 modulates the release of pain mediators caused by VEGF_165_b and oxaliplatin

To investigate the role of VEGF-A in pain signaling, we exposed organotypic slices to the selective blockers of VEGFR-1 (D16F7, 300 ​ng/mL) and VEGFR-2 (DC101, 10 ​ng/mL) and measured the quantity of the neuropeptides SP and CGRP released in the culture medium after 6 ​h of treatment with 10 ​μM oxaliplatin or 100 ​ng/mL VEGF_165_b. A 6-h time point was selected to capture early events before widespread damage, focusing on signaling mechanisms that precede injury, allowing clearer distinction of pro-nociceptive changes from neurotoxic damage.

**Fig. 9:**
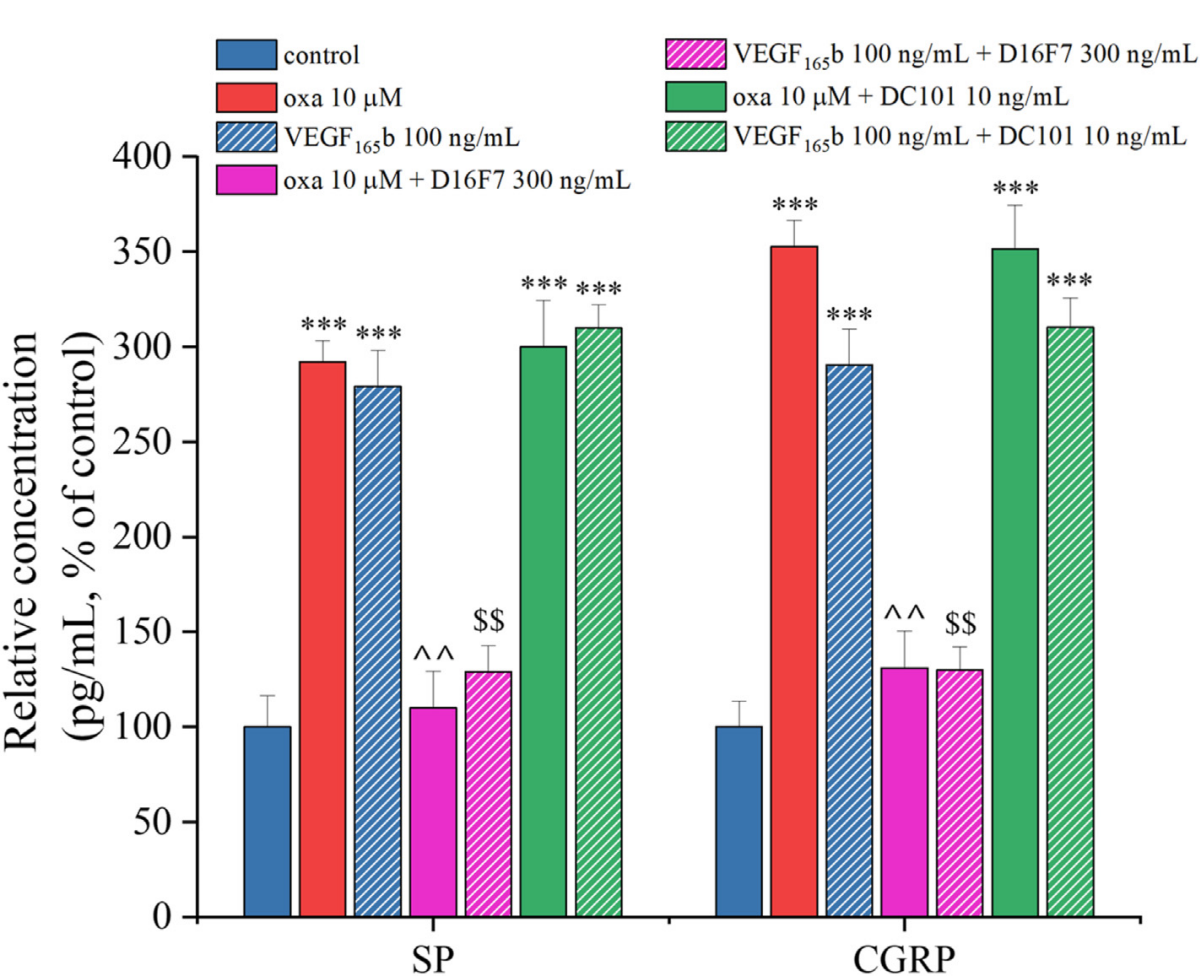
**VEGFR-1-mediates the release of CGRP and SP evoked by both oxaliplatin and VEGF**_**165**_**b.** The quantity of SP and CGRP released into the medium of the organotypic slices was measured by ELISA, after treatment with oxaliplatin (10 ​μM) or VEGF_165_b (100 ​ng/mL) for 6 ​h, alone or in the presence of the selective VEGFR-1 antagonist, D16F7 (300 ​ng/mL), or the selective VEGFR-2 antagonist, DC101 (10 ​ng/mL). Levels of the neuropeptides were normalized to cell protein concentration. Each value represents the mean ​± ​SEM of three independent experiments. ∗∗∗P ​< ​0.001 vs control; ˆ ˆ P ​< ​0.01 vs oxaliplatin treatment; ^$$^P ​< ​0.01 vs VEGF_165_b treatment.

Treatment with either oxaliplatin or VEGF_165_b caused a statistically significant increase in SP and CGRP release compared to control slices (Fig. 9). The co-treatment with D16F7, but not with DC101, reversed the increase in SP and CGRP release induced by oxaliplatin or VEGF_165_b (Fig. 9), indicating that the pain-related signaling is mediated by VEGFR-1.

**Fig. 10:**
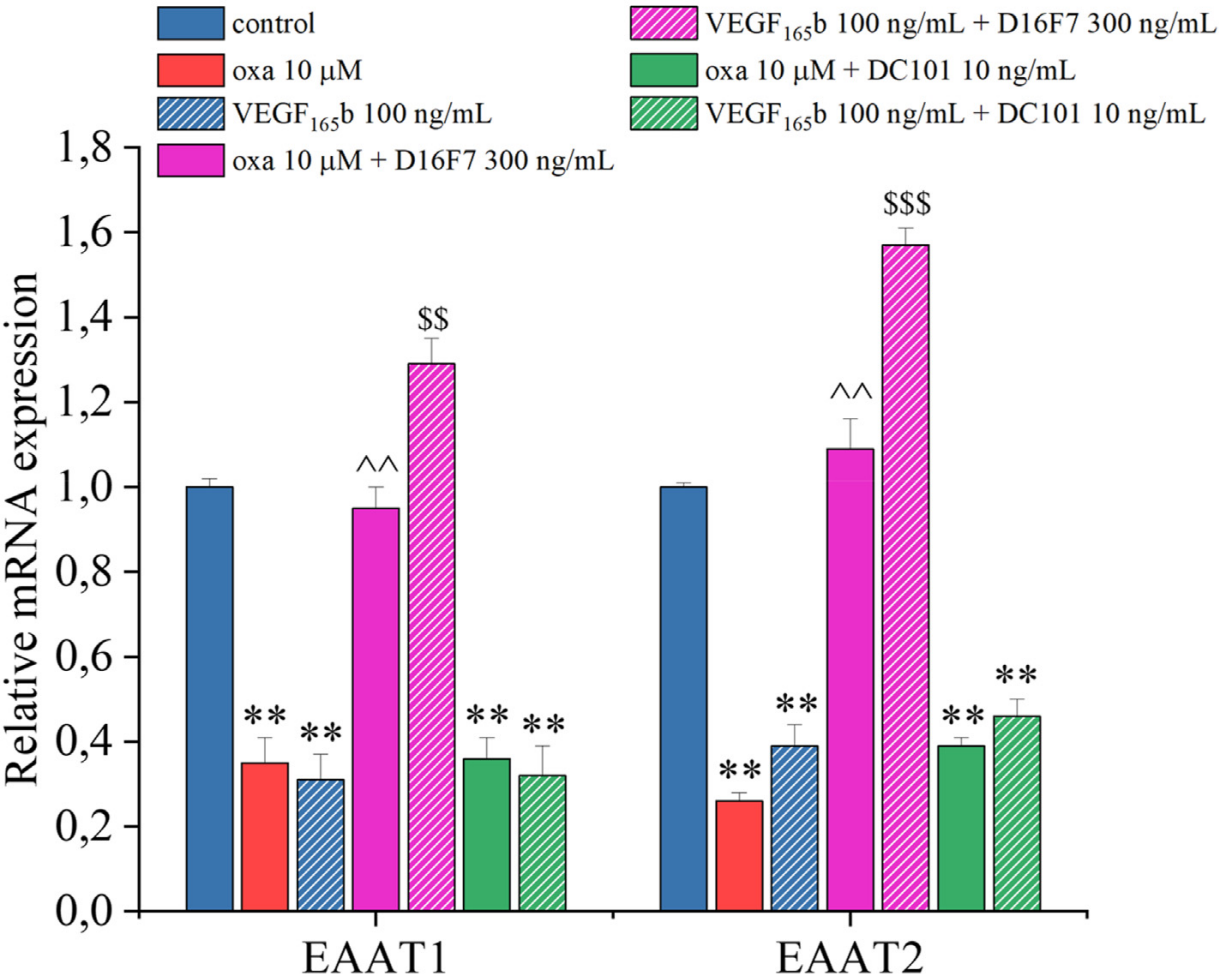
**VEGFR-1 mediates the downregulation of glutamate transporters evoked by both oxaliplatin and VEGF**_**165**_**b.** The mRNA expression of EAAT1 and EAAT2 genes was measured by RT-PCR, after treatment of organotypic spinal cord slices with oxaliplatin (10 ​μM) or VEGF_165_b (100 ​ng/mL) for 6 ​h, alone and in combination with the VEGFR-1 antagonist D16F7 (300 ​ng/mL) or the VEGFR-2 antagonist DC101 (10 ​ng/mL). Each value represents the mean ​± ​SEM of three independent experiments. ∗∗P ​< ​0.01 vs control; ˆ ˆ P ​< ​0.01 vs oxaliplatin treatment; ^$$^P ​< ​0.01 and ^$$$^P ​< ​0.001 vs VEGF_165_b treatment.

Given the pivotal role of glutamate as neurotransmitter, we currently explored changes in glutamate release by quantifying the expression of its transporters, the excitatory amino acid carrier (EAAT) 1 and 2, in the same experimental conditions and treatments indicated above. Treatment with either oxaliplatin or VEGF_165_b for 6 ​h reduced more than 50 ​% the expression of *EAAT1* and *EAAT2* genes compared to the control group (Fig. 10). Co-treatment with oxaliplatin and D16F7 returned *EAAT1* and *EAAT2* gene expression to control levels, while co-treatment with D16F7 and VEGF_165_b induced a statistically significant increase in the expression of both genes not only compared to the control but also compared to VEGF_165_b alone.

## Discussion

In the present study, we have demonstrated the dual role of VEGF-A, as pain-related and neuroprotective agent, by using an *in vitro* model of chemotherapy-induced neurotoxicity, achieved through distinct activation of VEGFR-1 and VEGFR-2, respectively.

Although CIN is triggered by dorsal root ganglion damage [30], several findings demonstrate a CNS impairment that leads to the central sensitization of pain [31,32]. Therefore, the spinal cord represents an interesting crossroad to study both morphological and functional alterations. By using rat organotypic spinal cord slices, we obtained an integrated *in vitro* system characterized by the maintenance of tissue cytoarchitectural organization and cell-to-cell contact, with an environment similar to that found *in vivo* [33].

In spinal cord slices, oxaliplatin evoked a concentration-dependent cytotoxicity, neuronal suffering, and astrocyte activation. Indeed, astrocytes appeared more elongated and with higher levels of GFAP, the main component of the intermediate filaments [34]. Accordingly, GFAP was found to be overexpressed in many *in vivo* models of CIN induced by oxaliplatin and others chemotherapeutic agents such as the proteasome inhibitor bortezomib and paclitaxel [[35], [36], [37], [38], [39]]. This astrocyte phenotype has been directly related to pain hypersensitivity [40,41] due to the release of pro-inflammatory cytokines (e.g., IL-1β), chemokines (e.g., MCP-1/CCL2), and growth factors (e.g., EGF, TGF-α, PDGF, VEGF) [42].

On the other hand, NeuN, a neuronal nuclear and perinuclear protein [43] was significantly decreased by oxaliplatin, similarly to what observed after ischemia or traumatic injury [[44], [45], [46], [47], [48]]. Additionally, oxaliplatin reduced the expression of glutamate transporters EAAT1 and EAAT2 [49,50], suggesting an impairment in the glutamate detoxification process, potentially leading to the accumulation of this algogenic and excitatory neurotransmitter in the synaptic cleft. Neuronal hyperactivity may trigger the release of neurotransmitters such as SP and CGRP, key mediators in the development and maintenance of pain [[51], [52], [53], [54]], as already observed *in vivo* [55].

According to our previous findings [10,24], oxaliplatin was found to increase VEGF-A release from astrocytes. Interestingly, the blockade of astrocyte activity by the aconitase inhibitor fluorocitrate (which undermines the Krebs Cycle) [56,57] resulted in increased oxaliplatin-induced cytotoxicity after 6 ​h of treatment. This damage was prevented by treating spinal cord slices with recombinant VEGF_165_b, which showed a concentration-dependent protective effect against oxaliplatin. VEGF_165_b was also able to reverse the alteration of neuronal and glial markers caused by oxaliplatin. These effects were specifically mediated by VEGFR-2, since they were mimicked by VEGF-E, the viral isoform of VEGF that acts as specific ligand of VEGFR-2 [58], and blocked by the specific anti-VEGFR-2 mAb DC101 [59].

The protective effects of VEGF-A presented here agree with its ability to safeguard stressed neurons in pathological conditions, inducing axon extension and branching, promoting synaptic plasticity and triggering astrocyte proliferation [16,60]. On the other hand, the reduced expression of VEGF-A caused the degeneration of motor neurons [61,62]. VEGF-A is known to be involved in the development of the nervous system, both in vessel differentiation and formation of the developing brain, as well as in neurogenesis and neuron growth control [62,63].

The relevance of VEGFR-2 mediated signaling in the neuroprotective properties of VEGF-A was confirmed in Schwann cells [64] and in dorsal root ganglion neurons injured by hyperglycemia [65] or by neurotoxic drug (paclitaxel) [66], through induction of Heat Shock Protein 90 deacetylation and increase of Bcl-2 [66]. Starting from these findings, Verheyen et al. (2013) emphasized that systemic anti-VEGF-A therapies may interfere with the neuroprotective activities of this growth factor, with important implications in cancer patients treated with such anti-angiogenic agents in combination with neurotoxic chemotherapy [66]. Interestingly, VEGFR-2 expression in spinal cord was enhanced by oxaliplatin *in vivo* [24] supporting the attempt to protect the nervous tissue, and its block with DC101 in our control organotypic slices increased neurotoxicity confirming the VEGFR-2 implication in the maintenance of nervous tissue homeostasis.

Our results show that VEGF_165_b enhanced the release of pain mediators like SP and CGRP, reduced glutamate transporters EAAT1 and EAAT2 mimicking the effects induced by oxaliplatin. The alteration of these parameters, when separately evoked by VEGF_165_b or oxaliplatin, were selectively prevented by the anti-VEGFR-1 D16F7 mAb. The anti-VEGFR-2 DC101 mAb was ineffective in modulating pain mediators in accordance with the lack of VEGFR-2 interference in oxaliplatin-induced pain shown *in vivo* [24]. To note, VEGFR-2 was implied in different pain conditions; the role of its binding protein neuropilin-1 was particularly highlighted [[67], [68], [69]].

As a molecular validation, these findings offer, *in vitro*, the mirror image of what observed *in vivo*.

Selvaraj and colleagues demonstrated that tumor-derived VEGF-A and PlGF-2 augmented pain sensitivity through the selective activation of VEGFR-1 expressed in sensory neurons in human cancer and mouse models [70]. Consistently, we demonstrated, *in vivo,* that D16F7 mAb blocked neuropathic pain induced by oxaliplatin, paclitaxel and vincristine [24]. While the study's results offer valuable insights into molecular mechanisms, caution is warranted in extrapolating these findings directly to human neurobiology and clinical conditions due to inherent variations between rat and human systems, potentially impacting the generalizability of the outcomes to human experiences with chemotherapy-induced neurotoxicity and pain.

In conclusion, this study demonstrates that VEGF-A amplifies pain related signals through VEGFR-1 and promotes neuroprotection through VEGFR-2. Our findings suggest that oxaliplatin-induced neurotoxicity may be managed by the selective blockade of VEGFR-1 that would allow the endogenous VEGF-A to carry out its neuroprotective effect through VEGFR-2.

## Ethics approval

Formal approval to conduct the study was obtained from the Italian Ministry of Health (No. 17.E9C.N.NOV), from the Animal Subjects Review Board of the University of Florence and from the Animal Ethics Committee of University of Campania of Naples.

## Consent for publication

Not applicable.

## Availability of data and material

After the publication the dataset are available from the corresponding author on reasonable request.

## Author contributions

Conceptualization, L.D.C.M., G.G.; Data curation, A.T., E.L., C.P.; Formal analysis, V.F., F.M., D.V.; Funding acquisition, C.G., G.G., P.M.L; Investigation, A.T., L.M.; Methodology, E.G., C.P., T.M.; Project administration, L.D.C.M; Resources, G.M., L.D.C.M.; Software, T.M., C.P., E.G.; Supervision, L.D.C.M., C.G., G.G., G.M. P.M.L; Validation, V.F., F.M., C.C., C.P.; Visualization; A.T., E.L., C.P., V.F., E.G.; Roles/Writing - original draft, L.D.C.M., A.T., E.L.; and Writing - review & editing L.M., T.M., G.G., P.M.L., E.G.

All the authors have read and approved the final version.

## Fundings

This work was supported by IMI2 project NeuroDerisk (ID: 821528), by KNOWPAIN, a grant funded by Tuscany region to C.G., and by National Recovery and Resilience Plan (NRRP), project MNESYS (PE00000006) – A Multiscale integrated approach to the study of the nervous system in health and disease (DN. 1553 October 11, 2022) to C.G. and G.G. In part this work was supported by #NEXTGENERATIONEU (NGEU) funded by the Italian Ministry of University and Research (MUR) and by the MUR PRIN 2022 (ID:2022EXSWZ2) to G.G.; and by the Italian Association for Cancer Research (AIRC) under IG 2022 (ID:27728) project and by the Italian Ministry of Health RC2023 to P.M.L.

## Declaration of competing interest

The authors declare that they have no competing interests.

## Figures and Tables

**Suppl. File 1:**
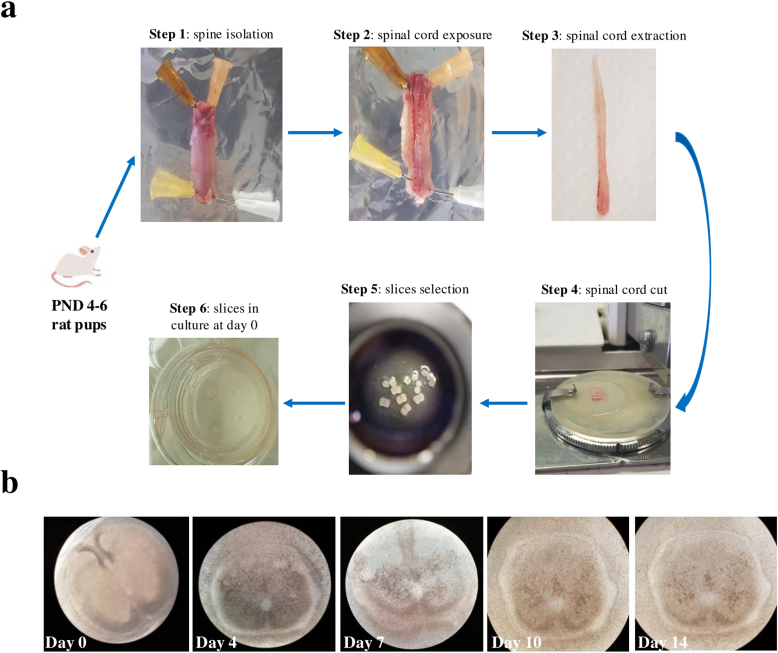
**Rat organotypic spinal cord slices obtaining**. The experimental protocol to obtain the organotypic spinal cord culture, starting from rat pups 4–6 post-natal days (a). Representative pictures of spinal cord slices during the 14 days of maturation, showing that structural integrity is maintained (b).

**Suppl. File 2:**
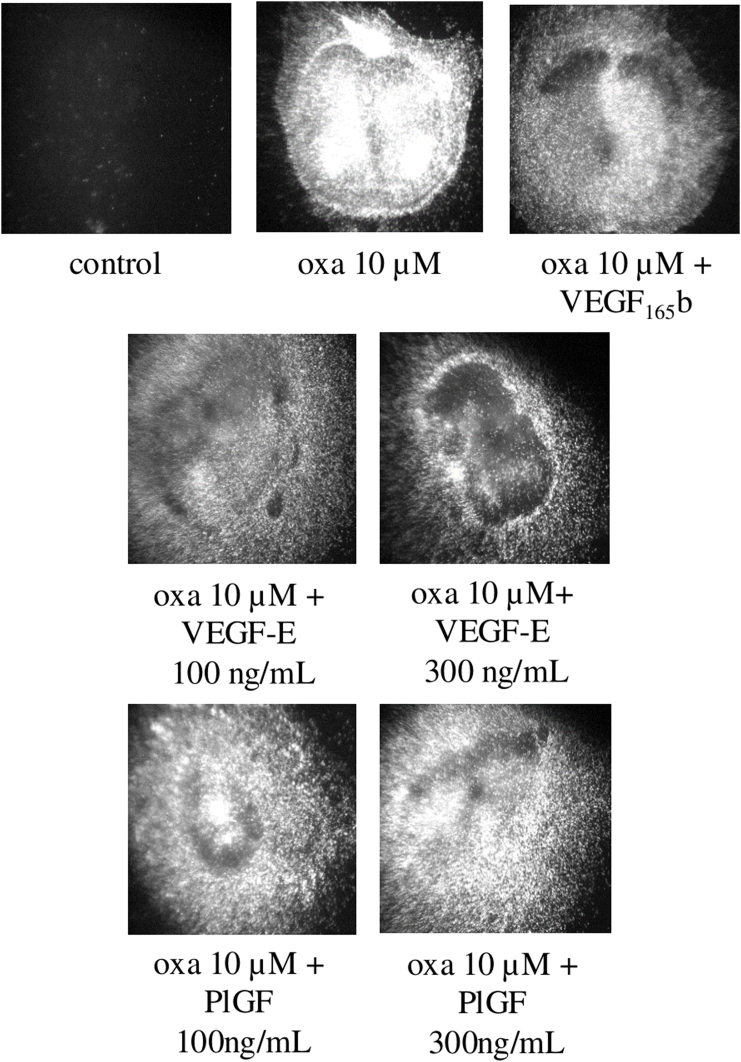
Representative pictures of the qualitative analyses of PI (5 ​μg/mL) fluorescence intensity, performed to evaluate the effect of the VEGFR-2 ligand, VEGF-E (100 and 300 ​ng/mL) and the VEGFR-1 ligand, PlGF (100 and 300 ​ng/mL) treatment on 10 ​μM oxaliplatin-induced toxicity.

**Suppl. File 3:**
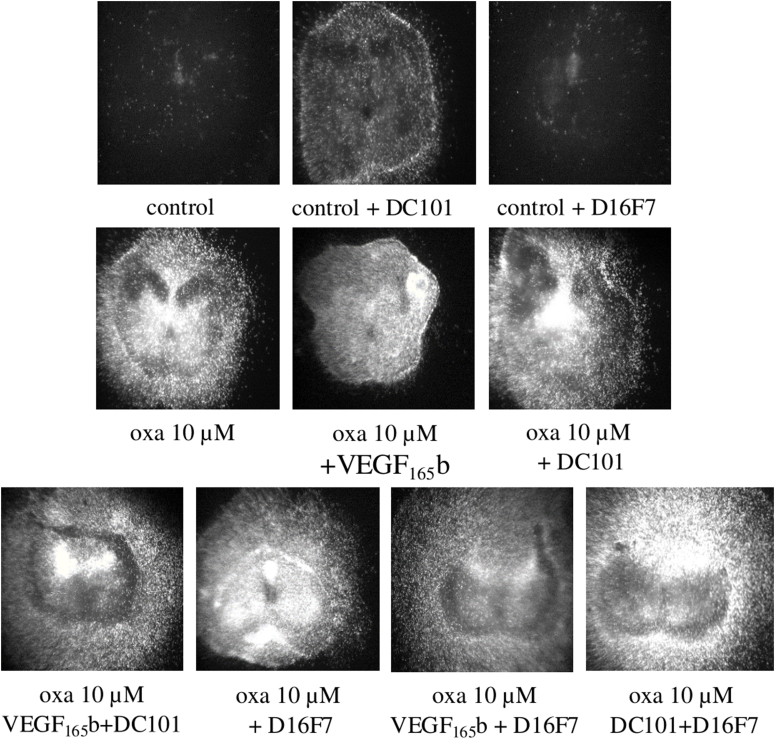
Representative pictures of the qualitative analyses of PI (5 ​μg/mL) fluorescence intensity, performed to evaluate the effect of the selective VEGFR-1 antagonist, D16F7 (300 ​ng/mL) or the selective VEGFR-2 antagonist, DC101 (10 ​ng/mL) mAbs treatment on 10 ​μM oxaliplatin-induced toxicity, in combination with VEGF_165_b (100 ​ng/mL).
